# Controlled Crystal Growth of All-Inorganic CsPbI_2.2_Br_0.8_ Thin Film via Additive Strategy for Air-Processed Efficient Outdoor/Indoor Perovskite Solar Cells

**DOI:** 10.3390/nano13192716

**Published:** 2023-10-06

**Authors:** Jitendra Bahadur, Jun Ryu, SungWon Cho, Saemon Yoon, Dong-Gun Lee, Dong-Won Kang, Padmini Pandey

**Affiliations:** 1 Department of Energy Systems Engineering, Chung-Ang University, Seoul 06974, Republic of Korea; jeetubaghel.567@gmail.com; 2 Department of Smart Cities, Chung-Ang University, Seoul 06974, Republic of Korea; jun2019@cau.ac.kr (J.R.); sungwon9605@cau.ac.kr (S.C.); saemony@cau.ac.kr (S.Y.); padawan1215@cau.ac.kr (D.-G.L.)

**Keywords:** additive strategy, all-inorganic perovskite solar cells, defect passivation, formamidinium bromide, outdoor/indoor photovoltaic performance

## Abstract

The evolution of defects during perovskite film fabrication deteriorates the overall film quality and adversely affects the device efficiency of perovskite solar cells (PSCs). We endeavored to control the formation of defects by applying an additive engineering strategy using FABr, which retards the crystal growth formation of CsPbI_2.2_Br_0.8_ perovskite by developing an intermediate phase at the initial stage. Improved crystalline and pinhole-free perovskite film with an optimal concentration of FABr-0.8M% additive was realized through crystallographic and microscopic analysis. Suppressed non-radiative recombination was observed through photoluminescence with an improved lifetime of 125 ns for FABr-0.8M% compared to the control film (83 ns). The champion device efficiency of 17.95% was attained for the FABr-0.8M% PSC, while 15.94% efficiency was achieved in the control PSC under air atmospheric conditions. Furthermore, an impressively high indoor performance of 31.22% was achieved for the FABr-0.8M% PSC under 3200 K (1000 lux) LED as compared to the control (23.15%). With a realistic approach of air processing and controlling the crystallization kinetics in wide-bandgap halide PSCs, this investigation paves the way for implementing additive engineering strategies to reduce defects in halide perovskites, which can further benefit efficiency enhancements in outdoor and indoor applications.

## 1. Introduction

Organic/inorganic hybrid lead halide-based perovskites have been considered emerging light harvesting materials due to their excellent optoelectronic properties such as long charge carrier diffusion length, strong light absorption, tunable optical band gap, high defect tolerance, and small exciton bonding energy, and facile fabrication process [1,2,3,4]. So far, the certified power conversion efficiency (PCE) of perovskite solar cells (PSCs) has rapidly improved from 3.8% [5] to 25.73% in a short span of time [6], competing with commercialized polycrystalline silicon (Si) solar cells [3]. However, hybrid PSCs exhibit poor long-term stability due to the volatile nature of organic components (e.g., methylammonium (MA) and formamidinium (FA)) and decomposition under high-temperature processing, which are the major obstacles for their commercialization [7,8,9]. To resolve these issues, the development of all-inorganic perovskites by substituting organic cations (MA^+^, FA^+^) in hybrid perovskites with inorganic cations (like cesium ion (Cs^+^)) [9,10] is a promising approach. The all-inorganic perovskites such as CsPbI_3_, CsPbI_2_Br, CsPbBrI_2_, CsPbBr_3_, and CsPbI_3-x_Br_x_ (x = 0 to 3) possess superior optoelectronic properties as well as outstanding physicochemical stability and tunable band gaps of 1.73 eV to 2.3 eV [11,12,13]. Among them, CsPbI_3_ has an appropriate optical band edge (~1.73 eV) to harvest the solar spectrum and is a favorable light-harvesting material to produce efficient all-inorganic PSCs [14]. However, the active α-CsPbI_3_ black inorganic perovskite phase easily converts into a non-perovskite yellow δ-phase at room temperature (RT) owing to phase transition [15]. On the other hand, CsPbBr_3_ has excellent phase stability, but its wider optical band gap (2.3 eV) limits the photovoltaic performance [16]. Thus, all-inorganic mixed-halide CsPbI_2_Br and CsPbI_2.25_Br_0.75_ perovskite compositions are promising candidates for preparing efficient single-junction PSCs and tandem solar cells (TSCs) due to their suitable band gap and good phase stability [17,18,19,20]. Nevertheless, the presence of various surface and bulk defects in solution-processed inorganic perovskites of poor crystallization leads to the formation of lower quality film, which limits the PCEs of inorganic PSCs [21,22]. Thus, the challenging task is to obtain highly crystalline, compact, and uniform inorganic perovskite thin films. In this regard, several strategies like interfacial engineering [23], metal doping [24], antisolvent engineering [25], halide ion substitution [26], thermal gradient annealing [27], surface regulation, and additive engineering [28] have been employed to improve perovskite film quality and the perovskite/charge transport layer (CTL) interface. Grain boundaries and surface defects in perovskite limit the CT dynamics at the interface and hence reduce the overall device PCE. A perovskite surface regulation strategy using silicon naphthalocyanine (Cl-SiNcTI) as an interlayer at the perovskite/Spiro-OMeTAD interface helps in limiting the deep-level defects and reduce non-radiative recombination. Zhou et al. reported a device PCE of 24.30% with over 240 h operational stability [29]. Li et al. introduced an n-type small molecule at the perovskite/PCBM interface with 23.57% device PCE of inverted PSC. In this surface regulation process, the polymeric molecule (PY-IT) significantly reduces the defect states and facilitates charge transport, while the polymeric linkers regulate the crystal grain orientation [30]. Yue et al. investigated the passivation of deep defect states and band energy alignment in perovskite by developing an in situ 2D perovskite phase on 3D perovskite, where diethylammonium iodide chemically interacted with lead iodide to form a 2D/3D phase [31]. Transition of the perovskite phase, ion migration, and degradation triggered due to residual tensile strain within perovskites limit the performance of PSCs. Li et al. investigated the reconstruction of perovskite surface and bulk through butylammonium cation (BAI) with dimethylformamide (DMF) post treatment [32]. In another study by Xu et al., residual strain in (CH(NH_2_)_2_)_0.95_Cs_0.05_PbI_3_ was regulated by introducing ligand-capped CsPbI_3_ quantum dots as an antisolvent, which reduced the lattice mismatching and controlled the crystal growth kinetics, eventually delivering over 23% device PCE [33].

Moreover, additive engineering is another approach that has been verified as an effective strategy for suppressing defects, producing high-quality inorganic perovskite film, improving phase stability, and increasing PCEs of the inorganic PSCs [34,35,36]. For instance, Patil et al. introduced ethylammonium bromide (EABr) as an additive into CsPbI_2_Br perovskite and suggested that EABr helps to retard the crystal growth, enlarge the grain size, and suppress the defect states, resulting in improved film quality [35]. They demonstrated that the EABr-assisted PSC exhibited a higher PCE of 14.47% than the reference device (12.62%). Zhang and his group added polyethylene glycol (PEG) into a CsPbI_2_Br precursor solution and obtained a device with 13.59% PCE [37]. The authors demonstrated that the addition of PEG modulates the energy level, retards the crystallization rate, and passivates the defect states of inorganic CsPbI_2_Br film, resulting in enhancement in the PSC’s performance. Jin et al. fabricated CsPbI_2_Br PSC with tetramethylammonium chloride (TMACl) additive and acquired a device PCE of 14.12% [38]. This group suggested that the incorporation of TMACl reduced the defect density and produced pinhole-free CsPbI_2_Br perovskite film with good crystallinity. Li and his co-workers incorporated 4-guanidinobenzoic-acidhydrochloride (4-GBACl) into a CsPbI_2_Br perovskite precursor [39]. They suggested that 4-GBACl significantly reduced the positive and negative charge defect states at the perovskite surface, leading to a device PCE of 15.59%. Fu et al. used 2-hydroxyethyl methacrylate (HEMA) as a liquid additive and achieved a device PCE of 16.13%, which is higher than the control PSC (14.10%) [40]. The authors demonstrated that HEMA additive improved the crystallization mechanism and passivated defects via the interaction of the C = O group of HEMA with undercoordinated Pb^2+^ ions, resulting in effectively suppressed charge recombination centers. Therefore, the additive strategy plays a key role in controlling crystallization kinetics and passivating the defects within the perovskite film, leading to high device performance.

Inspired by the above research studies, we introduced formamidinium bromide (FABr) with various concentrations (0.4, 0.8, and 1.2M%) into a CsPbI_2.2_Br_0.8_ perovskite precursor solution to investigate the effect of the FABr precursor additive on the crystallization kinetics, optoelectronic properties of thin films, film morphology, and device performance. We carried out a literature survey on CsPbI_2.2_Br_0.8_ perovskite compositions produced with the additive strategy, as shown in Table S1, and to the best of our knowledge, we are the first to use FABr as an additive in a CsPbI_2.2_Br_0.8_ perovskite precursor to produce a high-quality film. We observed that the FABr additive retards the crystallization and helps to control the crystal growth, resulting in improved surface morphology and crystallinity of the perovskite film. An optimum concentration of 0.8M% FABr significantly enhanced the film properties. As a result, the FABr-0.8M%-based device showed a remarkable *PCE* of 17.95%, which is higher than that of the control PSC (15.94%) under air atmospheric conditions (relative humidity of 30~40%, temperature of 20–24 °C). In addition, the FABr-0.8M% PSC exhibited a high indoor PCE of 31.22% compared to the control device (23.15%) under a 3200 K (1000 lux) LED lighting source. Thus, we have proposed a feasible method to develop a high-quality all-inorganic perovskite CsPbI_2.2_Br_0.8_ phase (wide band gap > 1.8 eV) under ambient conditions.

## 2. Materials and Methods

### 2.1. Materials

Indium-doped tin oxide (ITO)-coated glass slides (2 cm × 2 cm) with a sheet resistivity of 10 Ω/□ were obtained from AMG, Korea. The zinc acetate dihydrate (Zn(CH_3_CO)_2_∙2H_2_O, 99.99%), lead bromide (PbBr_2_, 99.99%), and 4-tert-butylpyridine (tBP) were purchased from Sigma Aldrich (St. Louis, MO, USA). Formamidinium bromide (FABr, 99.99%) was obtained from Greatcell Solar Materials Pvt Ltd. Lead iodide (PbI_2_, 99.99%) was acquired from TCI chemicals. Gold pellets (Au, 99.99%) and Poly(3-hexylthiophene-2,5-diyl) (P3HT) were bought from Rieke Metals Inc. (Lincoln, NE, USA). Cesium iodide (CsI, 99.99%) and tin oxide (SnO_2_, 15% in H_2_O) colloidal solutions were obtained from Alfa Aesar (Ward Hill, MA, USA). 2-methoxyethanol (2-ME, 99%), acetone (99%), ethanolamine (99%), dimethyl sulfoxide (DMSO, 99.8%), isopropanol (IPA, 99.5%), and dimethylformamide (DMF, 99.5%) were purchased from Samchun Chemical (Seoul, Republic of Korea). Phenyl-C_61_-butyric acid methyl ester (PC_61_BM, 99.5%) was bought from Organic Semiconductor Materials. Chlorobenzene (CB, 99% GR grade) was acquired from Wako Chemicals. All the received chemicals were used in the fabrication of PSCs without any further purification.

### 2.2. Preparation of Precursor Solutions 

The 1.2 M of CsPbI_2.2_Br0.8 perovskite solution was prepared by dissolving 0.3319 g of PbI2, 0.3118 g of CsI, and 0.1761 g of PbBr_2_ into DMSO:DMF (7:3 *v*/*v*) mixed solvent and then stirring for 12 h at room temperature (RT) in an N_2_-filled glovebox to form a transparent yellow-color solution. For the ZnO precursor solution, 2 mL of 2-ME and 61.7 μL of ethanolamine were added into 0.2195 g of Zn(CH_3_CO)_2_∙_2_H_2_O and then stirred at 60 °C for 2 h. The SnO_2_ solution was prepared by mixing 0.3 mL SnO_2_ colloidal solution into 2.7 mL deionized (DI) water and then stirring for 4 h at RT. To obtain P3HT solution, 1 mL CB and 20 μL CB were added into 10 mg P3HT and then stirred overnight at RT in an N_2_-filled glovebox. Finally, PC_61_BM solution was obtained by adding 1 mL CB into 20 mg PC_61_BM and then stirring for 12 h at RT in an N_2_-filled glovebox. The prepared ZnO, SnO_2_, and perovskite precursor solutions were filtered using a 0.2 μm hydrophilic syringe filter (Advantec, Taipei, Taiwan), and the PC_61_BM and P3HT precursor solutions were filtered using a 0.2 μm hydrophobic syringe filter (Advantec).

### 2.3. Fabrication of Perovskite Solar Cells

The procedure of fabrication of the PSCs is displayed in Figure S1. In detail, ITO glass slides were sequentially cleaned with acetone and isopropanol for 20 min in each solution using an ultrasonic bath sonicator. The cleaned ITO glass slides were dried at 95 °C for 30 min in an oven. Afterward, ITO substrates were cooled at RT, and then kept for 20 min under ultraviolet–ozone (UV/O_3_) treatment to increase the wettability of the surface and remove organic/inorganic impurities. The prepared SnO_2_ precursor solution was deposited on UV/O_3_-treated ITO substrates at 3000 rpm for 30 s and then annealed at 150 °C for 30 min. Thereafter, the ZnO precursor solution was spin-coated on the substrates at 5000 rpm for 30 s and heated at 170 °C for 30 min. For perovskite films, 80 μL of perovskite precursor solution was deposited at 1000 rpm and 3000 rpm for 10 s and 30 s, respectively. After spinning for 8 s, dynamic hot-air treatment was carried out using a hot-air gun (BOSCH (Gerlingen, Germany), GHG 630 DCE Hot Air Gun—0601 94C 740) at 230 °C from 8 to 22 s to promote crystallization. After completing the spin-coating process, perovskite-coated substrates were immediately transferred to a hotplate and then annealed at 240 °C for 10 min. Then, 50 μL of P3HT precursor solution was deposited at 3000 rpm for 30 s, and then sintered at 100 °C for 5 min. For electron-only devices (SCLC measurement), a PCBM precursor solution was deposited on perovskite-coated substrates at 3000 rpm for 30 s and then annealed at 100 °C for 5 min. Finally, 80 nm of gold (Au) metal electrode was evaporated using a thermal evaporator at ~3 × 10^−6^ Torr. The active area of the cell was 0.04 cm^2^, which was defined using a shadow mask.

### 2.4. Characterization Techniques

Various characterization techniques were used to analyze the structural and optoelectronic properties of thin films, device photovoltaic performance, and charge carrier dynamics phenomena in the PSCs. In detail, X-ray diffraction (XRD) patterns of the thin films were measured with a scan rate of 2 °/min through an X-ray diffractometer (Cu Kα radiation, λ = 1.54 Å, Bruker-AXS, D8-Advance (Billerica, MA, USA)). The top surface morphology of the thin films was captured using field emission scanning electron microscopy (FE-SEM; SIGMA 300, Carl Zeiss (Oberkochen, Germany)). The UV-visible spectra and steady-state photoluminescence patterns (with excitation wavelength of 495 nm) were recorded via ultraviolet-visible spectroscopy (UV-2700, Shimadzu (Kyoto, Japan)) and a spectrofluorometer (FP-8600, Jasco (Oklahoma City, OK, USA)), respectively. Time-resolved photoluminescence (TRPL) spectra were obtained with a fluorescence spectrometer (FlouTime 300, PicoQuant (Berlin, Germany)). The X-ray photoelectron spectroscopy (XPS) patterns were obtained using a K-Alpha X-ray Photoelectron Spectrometer (XPS) System (Thermo Fisher Scientific) with monochromatic Al Kα X-ray radiation (1486.6 eV).

The current density–voltage curves corresponding to the fabricated PSCs were recorded at a scan rate of 0.25 V/s under one-sun illumination (AM 1.5 G, 100 mW/cm^2^) via a xenon-lamp-based solar simulator (Peccell Technologies (Yokohama, Japan), PEC-L01). The light intensity of the xenon lamp was calibrated with a reference silicon solar cell (BS-500BK, Bunkoukeiki Co., Ltd., Osaka, Japan). The external quantum efficiency (EQE) patterns were measured with a CompactStat (Ivium Technologies (Eindhoven, The Netherlands); a xenon lamp 150 W power source, Abet Technologies (Milford, CT, USA), 13,014; and a monochromator (DongWoo Optron (Gwangju-Si, Republic of Korea), MonoRa500i). The indoor current density–voltage patterns were obtained under 3200 K light-emitting diode (LED) lighting conditions at 1000 lux. The input power density of 0.382 mW/cm^2^ at 1000 lux (3200 K LED) was used to calculate the indoor PCE. The transient photocurrent/photovoltage (TPC/TPV) decay curves were recorded with a multifunctional organic semiconductor parameter system (Mcscience (Suwon, Republic of Korea), T400). The electrochemical impedance spectroscopy (EIS) spectra were measured using a CompactStat (Ivium Technologies) by applying a bias voltage of 1.0 V under dark conditions. The space-charge-limited-current (SCLC) patterns were obtained for electron-only devices (ITO/SnO_2_/ZnO/perovskite/PC_61_BM/Au) using a Keithley-2400 source meter under dark conditions. The dark current density–voltage curves were measured using a CompactStat (Ivium Technologies). All the thin film and PSC measurements were conducted in ambient conditions (relative humidity of 30–40% and temperature of 20–24 °C).

## 3. Results and Discussion

### 3.1. Proposed Mechanism, Morphological, Structural, and Optoelectronic Properties of Films

**Figure 1:**
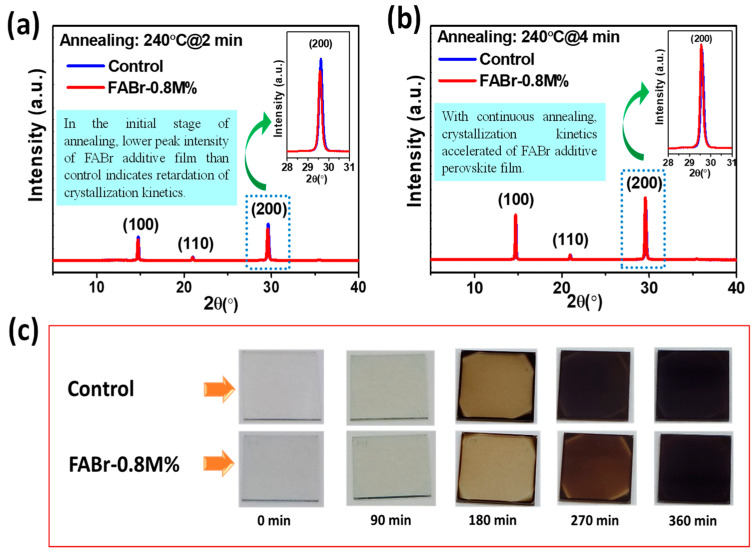
X-ray diffraction (XRD) pattern of control and FABr-0.8M% perovskite films after dynamic hot-air treatment followed by final annealing at (**a**) 240 °C for 2 min and (**b**) 4 min shows the crystal growth retardation and further acceleration in crystal growth with prolonged annealing at 240 °C. (**c**) Photographic illustration of control and FABr-0.8M% perovskite films at different time intervals from 0 min to 360 min during the crystal growth process.

We developed high-quality all-inorganic mixed-halide CsPbI_2.2_Br_0.8_ perovskite films by introducing different concentrations (0.04, 0.8, and 1.2M%) of FABr as a perovskite precursor additive. The dynamic hot-air method was used to develop perovskite films in ambient conditions at a relative humidity (RH) of 30–40% (atmospheric temperature of 20–24 °C). The as-prepared perovskite precursor solutions were deposited onto the ITO/ETL-coated substrates through spin coating as depicted in Figure S1, subsequently followed by hot-air treatment (230 °C) and final annealing at 240 °C (10 min). Here, we opted for the dynamic hot-air method, which is a feasible, non-vacuum, and antisolvent-free process for the fabrication of all-inorganic perovskite films under ambient conditions [41,42]. The complex intermediate phase (PbX_2_.DMSO:CsI) for CsPbI_2.2_Br_0.8_ was initially formed through the partial evaporation of polar solvent using dynamic hot-air treatment during the spinning process. Later, the intermediate phase was converted into a black CsPbI_2.2_Br_0.8_ perovskite active phase at 240 °C annealing for 10 min. Herein, the intermediate phase (PbX_2_.DMSO:CsI) plays a vital role in confining the initial number of nuclei centers through the specific evaporation rate of solvents [24,42]. The mechanism of the crystal growth kinetics of additive-assisted perovskite films can be observed through in situ absorbance, PL, and X-ray diffraction studies [43,44,45]. Here, to identify the role of the FABr additive on the crystal growth of perovskite film, both the control and FABr-0.8M% perovskite films were annealed at 240 °C at two different time intervals of 2 min and 4 min after dynamic hot-air treatment. The crystal growth of the perovskite film was further monitored through XRD as depicted in Figure 1a,b. At the primary stage after 2 min annealing (Figure 1a inset), the control film showed corresponding hkl planes of the crystalline perovskite phase.

The XRD peak showed a lower intensity for the FABr-0.8M% perovskite film compared to the control film (Figure 1a red line), indicating slower crystal growth kinetics in the FABr-0.8M% film. With continuous annealing for 4 min at 240 °C (Figure 1b), interestingly, the crystal growth kinetics of FABr-0.8M% accelerated further with improved crystallinity, as depicted in Figure 1b inset. We may speculate that the FABr in the perovskite precursor additive initially developed a CsI-FABr-DMSO:PbX_2_ intermediate phase, which retards perovskite crystal growth. To further identify the effect of the FABr additive on crystal growth retardation, both the control and FABr-0.8M% perovskite precursor were spin-coated on the substrate and kept a room temperature for several minutes to monitor the formation of back-phase CsPbI_2.2_Br_0.8_ perovskite, as depicted in Figure 1c. At the initial stage (0 min), completely transparent films were observed for both the control and FABr-0.8M% films; however, the conversion of perovskite film from transparent color to dark brown was reached earlier for the control film as compared to FABr-0.8M%, which indicates the effectiveness of FABr as an additive in retarding the crystallization of perovskite.

**Figure 2:**
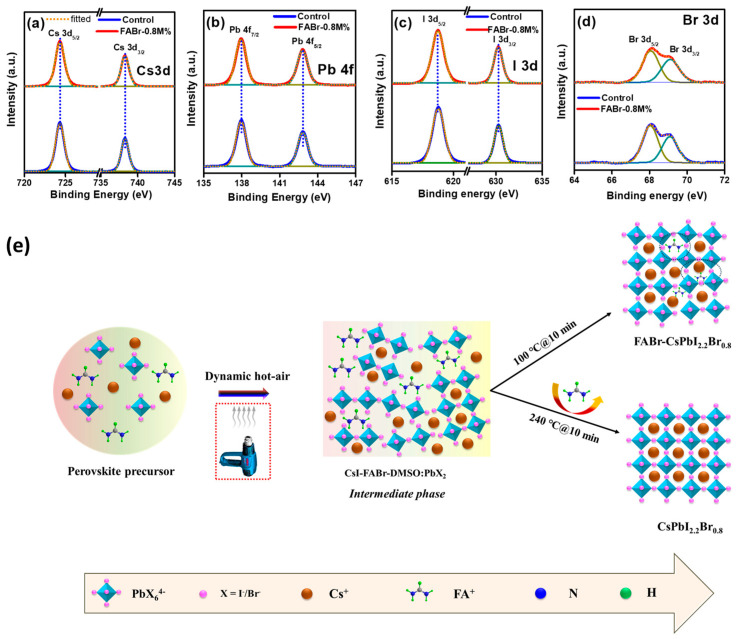
X-ray photon spectroscopy (XPS) core spectra: (**a**) Cs 3d core spectra, (**b**) Pb 4f, (**c**) I 3d, and (**d**) Br 3d of control (blue line) and FABr-0.8M% (red line), respectively. (**e**) Schematic representation of the plausible mechanism.

We presume that during annealing at 240 °C, FA^+^ cation sublimates from the final perovskite film and Cs^+^ cations fill the A-sites to develop a phase-pure CsPbI_2.2_Br_0.8_ perovskite film. In this line, XPS measurements were further conducted on control, 100 °C annealed FABr-0.8M% (FABr-0.8M%@ 100 °C), and 240 °C annealed FABr-0.8M% (FABr-0.8M%@240 °C or target) perovskite films to testify this statement. Figure S2a–e depict the XPS core spectra of Cs 3d, Pb 4f, I 3d, Br 3d, and N 1s FABr-0.8M%@100 °C and FABr-0.8M%@240 °C films. The binding energy (BE) peaks for the FABr-0.8M%@100 °C film appeared at 723.96 eV and 737.85 eV, assigned to Cs 3d_5/2_ and Cs 3d_3/2_ in the Cs core spectrum, respectively (Figure S2a), while a significant shift in the XPS BEs to 724.31 eV and 738.23 eV was observed in the case of FABr-0.8M%@240 °C (Figure S2a), which could be ascribed to the change in electron cloud density around the Cs^+^ cations through the incorporation of FA^+^ cations. As per the reported studies, a relevant shift in the binding energy of the Cs core spectrum was found due to incorporated cations in the A-site of the perovskite structure, such as with Li^+^, K^+^, Na^+^, and Rb^+^ [46,47]. We speculate that the presence of a FA^+^ cation would participate in perovskite crystal formation by partially occupying the A-site cation vacancies at 100 °C for 10 min as shown in the schematic diagram (Figure 2e). At low-temperature processing (100 °C for 10 min), a few cation positions can be filled by FA^+^, and existing Cs^+^ cations would be repelled by the presence of FA^+^ ions due to the same polarity of charges (as shown in the schematic diagram (Figure 2e)) that can change the electron cloud density near Cs^+^ cations in the resulting perovskite structure. As a result, occupancy of A-site cations position by FA^+^ ions and repulsive phenomena between FA^+^ and Cs^+^ cations might introduce the shift in the Cs core spectra at low-temperature processing (100 °C for 10 min). As for the high-temperature annealing at 240 °C, on the other hand, the completely evaporated FA+ ions could not cause such a shift in the Cs spectrum, as shown in Figure S2a. However, similar BE positions were observed for Pb at 137.99 eV (4f_7/2_) and 142.82 eV (4f_5/2_), I at 618.76 eV (3d_5/2_) and 630.23 eV (3d_3/2_), and Br at 68.09 eV (3d_5/2_) and 69.09 eV (3d_3/2_), as shown in Figure S2. Different core spectra of control perovskite, i.e., Cs 3d, Pb 4f, I 3d, and Br 3d, were compared with FABr-0.8M% (target) film as depicted in Figure 2a–d, with no shift in the BEs. Specifically, similar BE peak positions of the Cs core spectra for control and FABr-0.8M% (Figure 2a) suggest that during 240 °C annealing, the FA^+^ cation sublimates from the final perovskite film and the Cs^+^ cation acquires the A-site position, leading to the formation of pure-phase perovskite. This was further evident from N 1s core spectra (Figure S2e), where a BE signal was detected at 400.06 eV for FABr-0.8M%@100 °C while no such peak was observed for FABr-0.8M%@240 °C film. We also measured the XPS spectra of FABr-0.8M%-based perovskite film after etching the surface to ~100 nm in depth and compared it with the XPS spectra of a surface scan of the FABr-0.8M%-based perovskite film, as shown in Figure S3. Notably, in the case of the etched surface scan of the XPS measurement, no binding energy peak signal was detected in the N 1s core spectra (Figure S3e), which also confirmed that FA^+^ cations completely sublimated at constant high-temperature annealing (240 °C for 10 min) under ambient conditions. Moreover, it has been reported that ethylammonium bromide (EABr) and formamidinium chloride (FACl) as additives were used to improve the quality of inorganic perovskite (CsPbI_2_Br) film and it was suggested that organic cations completely sublimated during the high-temperature constant thermal annealing process under ambient air [35,48]. In our previous research study, we also introduced phenethylammonium iodide (PEAI) as an additive to construct a high-quality inorganic perovskite (CsPbI_2_Br) film and found that the organic cation (PEA^+^) completely sublimated during the high-temperature sintering process under ambient conditions [22].

Based on the experimental findings as discussed above, a plausible mechanism is shown in Figure 2e, where at the initial stage the perovskite precursor with FABr additive was spin-coated on the substrate, followed by dynamic hot-air treatment at the 8th second of spinning. The dynamic hot-air treatment helped in controlling the nucleation sites and eventually a CsI-FABr-DMSO:PbX_2_ intermediate phase developed. Thus, the formed complex could help in crystal growth retardation to develop highly crystalline perovskite film. Meanwhile, with constant annealing at a temperature of 240 °C, the FA^+^ cation sublimated from the perovskite film followed by the construction of a pure CsPbI_2.2_Br_0.8_ perovskite phase.

**Figure 3:**
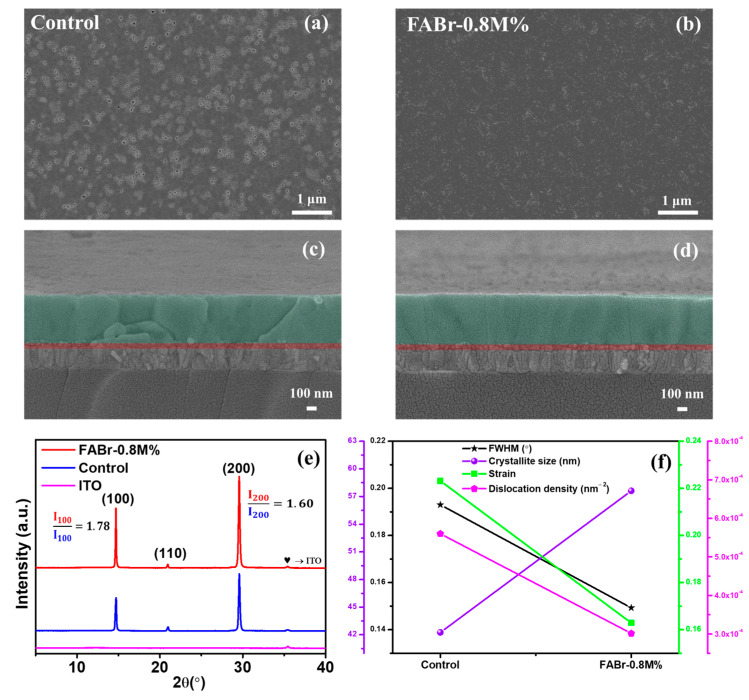
(**a**,**b**) Field-emission scanning electron microscopic (FESEM) surface view and (**c**,**d**) cross-sectional images of control and FABr-0.8M%, respectively. (**e**) XRD patterns of control and FABr-0.8M% perovskite films. (**f**) Extracted FWHM through XRD, calculated crystallite size, strain, and dislocation density comparison of control and FABr-0.8M% perovskite films.

FESEM characterization was conducted to explore the impact of the FABr additive on the morphology of the perovskite film, and Figure 3a,b depict the FESEM surface micrographs of the control and FABr-0.8M% films, respectively. A poor surface morphology with several pinholes can be easily noticeable in the control perovskite film, which acts as a defect state. Additionally, random crystal growth with excessive grain boundaries was observed through the cross-sectional view (Figure 3c), which formed due to rather quick crystal growth. With adding 0.8M% FABr additive into the perovskite precursor, the surface morphology improved with a significant reduction in pinholes, which indicates the FABr additive method controls the crystal growth process and leads to the formation of perovskite film with suppressed trap densities (Figure 3b). Also, the cross-sectional FESEM view (Figure 3d) shows controlled crystal growth with limited grain boundaries. It is further established that FABr as a perovskite additive controls the chemical reaction among CsI-FABr-DMSO:PbX_2_, resulting in crystal growth retardation that assists the Ostwald ripening. Prolonged crystal growth in FABr-0.8M% further improves the perovskite film morphology with larger grain size and reduced pinholes.

The X-ray diffraction (XRD) patterns for the control and FABr-0.8M% are shown in Figure 3e, and were used to determine the effect of FABr on the crystallinity of perovskite. The characteristic XRD peaks that appeared at 14.78°, 21.01°, and 29.66° correspond to the (100), (110), and (200) hkl planes, respectively, confirming the formation of α phase CsPbI_2.2_Br_0.8_ perovskite [21]. Notably, no impurity phase was detected in the FABr-0.8M% film, and the FABr-additive-assisted perovskite film revealed comparatively high-intensity crystalline peaks, which confirmed that the addition of FABr influences the crystal growth kinetics of perovskite. The detailed crystallographic parameters, i.e., full width at half maxima (FWHM), and the crystallite size (D), strain (ε), and dislocation density (δ), were calculated from the XRD data as shown in Figure 3f and Table S2. The average crystallite size was calculated using the Debye–Scherrer Equation (1) [36,49]:

(1) $$D=\frac{k\lambda }{\beta cos\theta }$$

where k is the shape factor, λ is the wavelength of Cu-Kα (1.54 Å), β represents the FWHM (degree), and θ is the diffraction angle (degree). Micro-strain (ε) was extracted from the following Hall–Williamson equation [36]:

(2) $$\beta cos\theta =\frac{k\lambda }{D}+4ɛsin\theta$$

Thus, the value of ε can be estimated as [50]

(3) $$ɛ=\frac{\beta }{4tan\theta }$$

and the dislocation density can be estimated from the equation below [41]:

(4) $$\delta =\frac{n}{D^{2}}$$

where n is almost unity for minimal dislocation density.

The FWHM for FABr-0.8M% reduced to 0.1493 (from control (0.1930)), with an increase in the average crystallite size of 57.61 nm in comparison to that of the control film of 42.26 nm (Figure 3f), which indicates improved crystallinity of the FABr-0.8M% film. The micro-strain (ε) in solution-processed perovskite films is realized due to the existence of extended defects such as crystal imperfection, grain boundary defects, and so on. However, we noticed a significant reduction in strain and dislocation density for the FABr-0.8M% film, indicating a substantial reduction in defect density.

**Figure 4:**
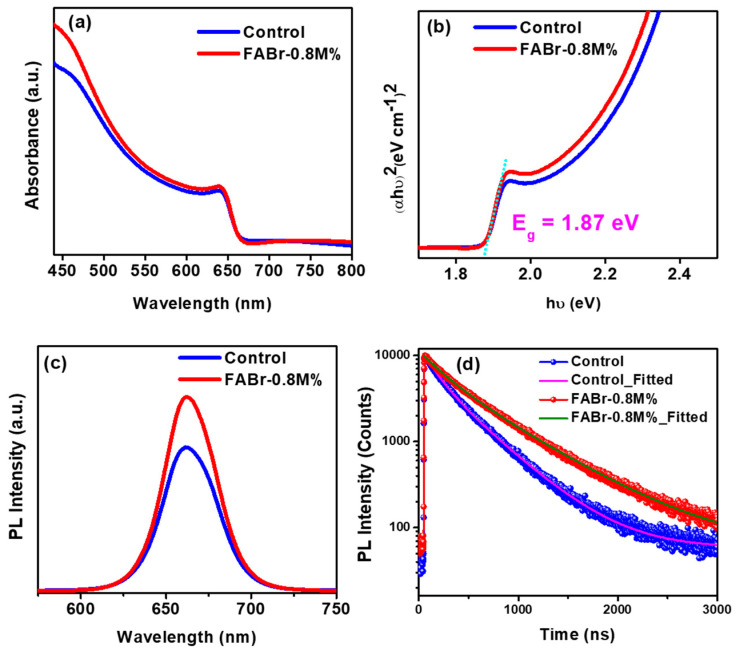
(**a**,**b**) UV-visible absorbance and calculated bandgap of control and FABr-0.8M% (target) perovskite film. (**c**) Steady-state PL and (**d**) time-resolved PL (TRPL) of control and FABr-0.8M% perovskite film.

The UV-Vis spectroscopic analysis was further conducted on the control and FABr-0.8M% perovskite films to identify the absorption and associated bandgap. Figure 4a shows the absorption spectra of the control and FABr-0.8M% perovskite films in the wavelength range of 440 to 800 nm. We may attribute the slight enhancement in the absorption signal (FABr-0.8M%) to the improvement in perovskite film morphology [35]. The bandgap value was further calculated through the Tauc plot, and the estimated bandgap is around 1.87 eV (as shown in Figure 4b), which suggests that FABr does not alter the bandgap of final perovskite. The existence of surface and bulk defects in halide perovskites deteriorates the device efficiencies through non-radiative recombination channels. The steady-state (SS) and time-resolved photoluminescence (TRPL) spectroscopic analyses were further conducted on the control and target perovskite films. Figure 4c depicts the SSPL curve of the FABr-0.8M% perovskite film in comparison with the control film at an excitation wavelength of 495 nm. It is noteworthy to mention that the enhanced PL signal in the FABr-0.8M% perovskite film is attributed to defect passivation with suppressed non-radiative recombination. To verify the SSPL results, we conducted TRPL measurements for control and FABr-0.8M% perovskite films on the glass substrates, as shown in Figure 4d. The curves were fitted biexponentially using the following Equation (5) [40,51] and the lifetime parameters are listed in Table S3.

(5) $$F\left(t\right)=A_{1}\exp\left(-\frac{t}{\tau_{1}}\right)+A_{2}\exp\left(-\frac{t}{\tau_{2}}\right)$$

where *τ_1_* and *τ_2_* represent bulk-defect-assisted radiative recombination and surface-trap-induced non-radiative recombination, respectively, and *A_1_* and *A_2_* are related amplitudes.

The average lifetime (*τ_avg_*) corresponding to perovskite films was estimated using the following equation [52]:

(6) $$\tau_{avg}=\frac{{{{A_{1}\tau }_{1}^{2}+A}_{2}\tau }_{2}^{2}}{A_{1}\tau_{1}+A_{2}\tau_{2}}$$

The average lifetime is 83 ns and 125 ns for the control and target perovskite films, respectively. The enhancement in carrier lifetime (TRPL) and PL intensity in SSPL signifies the suppressed defect densities in the FABr-0.8M% additive-assisted perovskite film.

### 3.2. Investigation of PV Parameters, Defect States, and Charge Carrier Dynamics of PSCs

**Figure 5:**
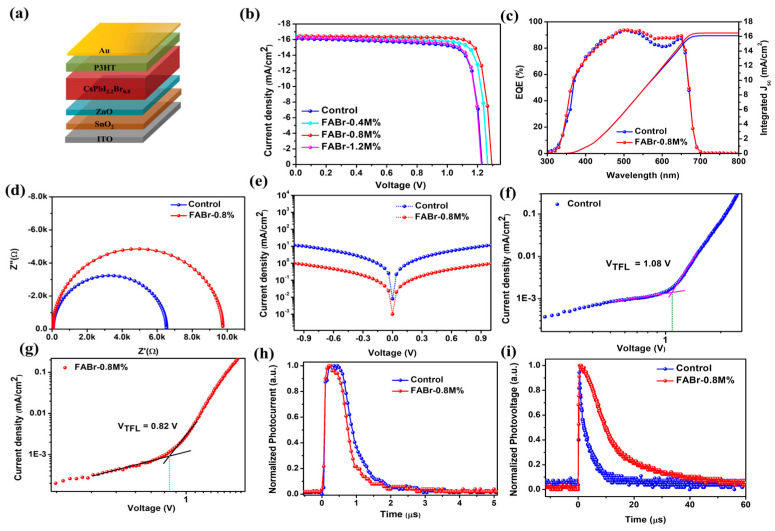
(**a**) Schematic diagram of n-i-p device structure. (**b**) Current density vs. voltage curve of control and FABr-0.8M%-based PSC. (**c**) External quantum efficiency curve and corresponding integrated current density of control (blue line) and FABr-0.8M% (red line). (**d**) Dark J-V curves for identifying leakage current for control and FABr-0.8M% devices. (**e**,**f**) Trap density calculated through hole-only device for control (blue line) and FABr-0.8M% (red line), respectively. (**g**) Electrochemical impedance spectroscopy graphs. (**h**,**i**) Transient photocurrent and photovoltage for control and FABr-0.8M% devices.

To examine the impact of FABr additive on the photovoltaic performance, we fabricated the PSCs with a device architecture of ITO/SnO_2_/ZnO/Perovskite/P3HT/Au as shown in Figure 5a. The measured current density–voltage (J-V) characteristics of fabricated PSCs (control, 0.4, 0.8, and 1.2M% FABr additive) are shown in Figure S4 (reverse and forward scans) and Figure 5b (reverse scan) and correspond to the obtained photovoltaic (*PV*) parameters tabulated in Table S4 and Table 1, respectively. The control PSC exhibited a power conversion efficiency (*PCE*) of 15.94% with a short current density (*J_sc_*) of 16.10 mA/cm^2^, an open-circuit voltage (*V_oc_*) of 1230 mV, and fill factor (*FF*) of 80.51%. After the addition of FABr-0.4M%, the photovoltaic performance of the device was significantly enhanced, exhibiting a PCE of 17.12% with *J_sc_* of 16.42 mA/cm^2^, *V_oc_* of 1265 mV, and *FF* of 82.44%. When the FABr concentration increased to 0.8M%, the *PV* parameters of the PSC further improved, exhibiting a *PCE* of 17.95% with *J_sc_* of 16.46 mA/cm^2^, *V_oc_* of 1293 mV, and *FF* of 84.36%. Such notable improvements in the *PV* parameters after the addition of FABr are mainly due to better surface morphology, enhanced crystallinity, and reduced surface defects of the perovskite layer as demonstrated above. When the FABr concentration increased to 1.2M%, the device *PCE* decreased to 16.23%, which indicates that an optimum concentration of FABr additive is 0.8M%. From a systematic literature study (Table S1), it is worth noting that the optimized device (FABr-0.8M%) exhibited one of the highest *PCEs* in the case of the CsPbI_2.2_Br_0.8_ PSCs. The external quantum efficiency (EQE) curves of the control and FABr-0.8%-based PSCs are displayed in Figure 5c. From EQE spectra, the integrated *J_sc_* values for control and FABr-0.8%-based devices were calculated to be 16.07 mA/cm^2^ and 16.44 mA/cm^2^, respectively, which are in good agreement with the obtained *J_sc_* values from J-V curves. In addition, reproducibility is an important parameter for the commercialization aspect of devices. We fabricated 20 independent devices of each condition (control and FABr-0.8M%), and *PCE* histograms are displayed in Figure S5. The FABr-0.8M%-based PSCs exhibited a narrow distribution of *PCEs* as compared to control devices, confirming that the proposed FABr-based additive strategy has a good reproducibility.

As depicted in Figure 5d, the FABr-0.8M%-based PSC exhibited a lower dark current value than the control device, suggesting that the FABr additive reduces the leakage current, which is attributed to the presence of a defective surface and pinholes [39] according to the following equation [52]:

(7) $$V_{oc}=\frac{K_{B}T}{q}\ln\left(\frac{J_{sc}}{J_{dark}}\right)$$

where *K_B_*, *T*, *q*, and *J_dark_* are Boltzmann constant, absolute temperature (Kelvin), electrical charge, and dark current density, respectively. From Equation (7), the value of *V_oc_* is proportional to the logarithmic ratio of *J_sc_* and *J_dark_*. The FABr-0.8M% PSC showed an increment in *J_sc_* and decrement in *J_dark_*, leading to a high *V_oc_* of 1293 mV, indicating that charge trap states were significantly reduced with the optimized FABr additive.

To quantitively evaluate the defect states of the control and FABr-0.8% assisted perovskite films, we opted for the space-charge-limited-current (SCLC) method and measured I-V curves by fabricating electron-only devices with the architecture of ITO/SnO_2_/ZnO/perovskite/PCBM/Ag. From the obtained spectra, the value of trap-filled voltage (*V_TFL_*) was extracted at the kink point, indicating the transition of the graph from the ohmic to trap-filled voltage region (non-linear) [3]. As shown in Figure 5e,f, the trap-filled voltage (*V_TFL_*) values were extracted to be 1.08 V and 0.82 V, corresponding to the control and FABr-0.8M% PSCs, respectively. The trap state density (*n_t_*) can be determined using the following formula [3]:

(8) $$n_{t}=\frac{2ɛ{ɛ}_{o}V_{TFL}}{eL^{2}}$$

where ε_o_, ε, e, and *L* are vacuum permittivity, relative dielectric constant of perovskite, electrical charge, and thickness of perovskite film, respectively. From Equation (8), the value of *n_t_* was determined to be 7.03 × 10^15^/cm^3^ and 5.33 × 10^15^/cm^3^ for the control and FABr-0.8M% based perovskite films. Noticeably, the optimized (FABr-0.8M%) perovskite layer exhibited a lower value of trap states as compared to the control film, revealing suppressed trap-assisted non-radiative recombination. A reduction in trap states is beneficial for mitigating the energy losses in PSCs. Energy loss (*E_loss_*) was estimated according to the following equation [53]:

(9) $$E_{loss}=E_{g}-{eV}_{oc}$$

As depicted in Figure S6, the FABr-0.8M%-based PSC showed low energy losses as compared to the control device, which is attributed to improved perovskite film quality.

To investigate the influence of the FABr additive on charge carrier dynamics, we recorded the electrochemical impedance spectroscopy (EIS) patterns under dark conditions with a basing voltage of 1.0 V and a frequency range of 2 MHz to 10 Hz (Figure 5g). According to the electronic circuit as shown in the inset of Figure 5g, the EIS patterns were fitted using Z-view software and obtained Nyquist parameters displayed in Table S5. The electronic circuit consists of series resistance (R_s_), recombinational resistance (R_rec_), and chemical capacitance (C_rec_) [3,54]. The Rs components in the PSCs are related to the metal electrode interface, wires, and ITO electrode interfaces [55,56]. The control and FABr-0.8M% PSCs showed almost comparable Rs owing to their similar device architectures. Moreover, the R_rec_ of the PSCs suggests that the recombination sites were at the electron transport layer (ETL) and perovskite interface [57,58]. Noticeably, the FABr-0.8M%-based PSC exhibited a higher value of R_rec_ (9706 Ω) than the control device (6478 Ω), suggesting that the charge carrier recombination was effectively reduced due to passivation of bulk and surface trap states in the FABr additive strategy, which is consistent with SCLC results.

Moreover, transient photocurrent/photovoltage (TPC/TPV) measurements were conducted to examine the charge carrier transport and recombination kinetics within the PSCs. As shown in Figure 5h (TPC decay), the charge carrier transport lifetime (τ_ct_) of the control and FABr-0.8M%-based PSCs were estimated to be 0.66 μs and 0.52 μs, respectively. Notably, the FABr-0.8M%-treated device exhibited a rapid decay time (0.52 μs) as compared to the control PSC (0.66 μs), confirming more efficient charge carrier extraction and transportation phenomena. Meanwhile, the TPV results (Figure 5i) demonstrated that the charge carrier recombination lifetime (τ_rec_) of 3.70 μs (control device) was increased to 10.64 μs (FABr-0.8M%-based PSC), which means that the trap-assisted recombination sites considerably decreased, which is in good agreement with TRPL and EIS results.

### 3.3. Investigation of Indoor PV Parameters and Long-Term Stability of PSCs

**Figure 6:**
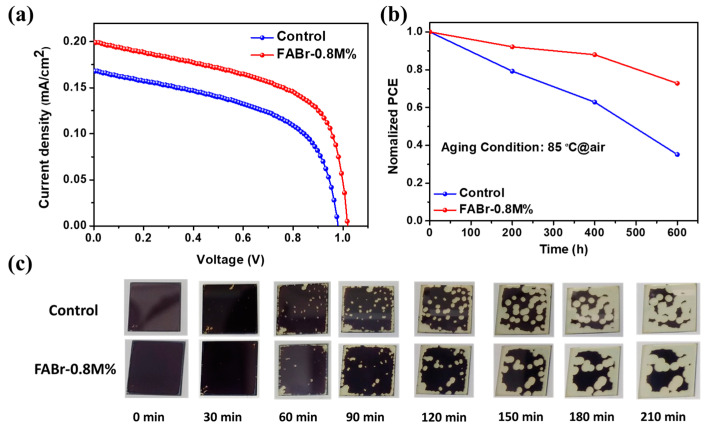
(**a**) Indoor current density vs. voltage curves for control and FABr-assisted PSCs, (**b**) long-term thermal stability of control and FABr-based PSCs after aging at 85 °C for 600 h (RH ~10–20% and temperature ~20–28 °C), and (**c**) digital photographs of control and FABr-0.8M%-based perovskite films at different time intervals with continuous aging under ambient conditions (RH ~30–40% and temperature ~20–24 °C).

In the current scenario, indoor photovoltaic technology has gained huge attention due to the development of internet of things (IoT)-based applications. Indoor PSCs are considered to be a promising energy resource to power the electronic units of IoT-based systems. In this regard, we measured the J-V curves of the control and FABr-0.8M% PSCs under indoor lighting conditions (LED, 3200 K, 1000 lux), and the obtained patterns are depicted in Figure 6a and related *PV* parameters are tabulated in Table 2. The input power of 0.382 mW/cm^2^ corresponding to 3200 K at 1000 lux was used to determine the indoor PCE of the PSCs. The control device exhibited an indoor *PCE* of 23.15% with *J_sc_* of 169 μA/cm^2^, *V_oc_* of 980 mV, and *FF* of 53.12%. With the addition of optimized FABr (0.8M%), the PSC’s indoor *PCE* was dramatically improved to 31.22% with *J_sc_* of 201 μA/cm^2^, *V_oc_* of 1031 mV, and *FF* of 57.27%. The power densities of the control and FABr-0.8M%-based PSCs were calculated to be 88 μW/cm^2^ and 118.7 μW/cm^2^, respectively. This significant improvement in indoor *PV* parameters is mainly due to decreased trap-assisted charge carrier recombination. In addition, the optimized indoor PSC (FABr-0.8M%) can be applied to power various portable electronic devices such as radiofrequency identification, LoRa backscatters, wrist watches, calculators, quartz oscillators, and hearing aids, as displayed in Figure S7. Additionally, we measured the indoor photovoltaic performance at 1500 lux and 2000 lux as shown in Figure S8. The input powers of 0.573 mW/cm^2^ and 0.764 mW/cm^2^ related to 1500 lux and 2000 lux (3200 K LED lighting conditions), respectively, were used to determine the PCE of the FABr-0.8M%-based PSC. The *PCEs* of 31.77% and 31.92% were found to correspond to 1500 lux and 2000 lux, respectively. It was observed that the FF of the PSCs under low lighting conditions is low. The photovoltaic performance of the PSCs decreases under indoor and outdoor lighting conditions due to the presence of various power losses such as ohmic loss, nonradiative recombination loss, and optical loss [59,60]. It has been demonstrated that the photovoltaic performance of PSCs under low lighting conditions could deteriorate owing mainly to two major reasons: ohmic losses and charge carrier recombination losses [59]. Moreover, it was found that ohmic losses in devices under indoor light conditions are mainly caused by shunt loss, while under outdoor light conditions the ohmic loss in devices mainly originates from series loss [59,61]. Therefore, devices could reveal different responses under indoor and outdoor lighting conditions. In addition, it is widely reported that a drop in FF value is due to a lower value of parallel parasitic resistance (Rp) [62,63]. Thus, we can speculate that a decrement in FF value might be due to the low value of Rp.

Finally, the long-term thermal stability of the unencapsulated control and FABr-0.8M%-based PSCs was examined by keeping devices at 85 °C under air conditions (RH~10–20% and temperature 20–28 °C) and obtained results demonstrated in Figure 6b. The presence of pinholes, voids, defective surface, grain boundaries, etc., is the main cause of poor structural stability of perovskite film because these surface defects allow moisture intrusion and permeation, resulting in perovskite active α-phase (black) conversion to non-perovskite yellow δ-phase [40,64]. From Figure 6b, the control PSC retained ~35.11% of the initial PCE value, whereas the FABr-0.8M%-assisted devices exhibited ~72.30% of the original PCE after aging for 600 h at 85 °C. Promisingly, the FABr-0.8M%-based PSC exhibited higher thermal stability than the control device. After optimizing the FABr-0.8M% additive, a noticeable enhancement in thermal stability was observed, which resulted in an ameliorated film quality. Moreover, we also investigated the air stability of the control and FABr-0.8M%-treated perovskite films under ambient conditions (RH ~30–40% and temperature 20–24 °C). The perovskite films’ degradation process was monitored by capturing the digital photographs displayed in Figure 6c. Interestingly, the FABr-0.8M%-assisted perovskite film showed better stability in ambient air as compared to the control film after aging for 210 min, which is due to enhanced crystallinity and dense morphology. Therefore, our findings suggested that an optimized concentration of FABr additive effectively healed bulk and surface defects by improving the film morphology, crystallinity, and controlled crystal growth mechanism, resulting in increased photovoltaic performance. Thus, the proposed FABr-based additive strategy can be a promising route for the further development of efficient and stable outdoor/indoor all-inorganic PSCs for practical applications.

## 4. Conclusions

In summary, we have successfully introduced an effective strategy using a FABr additive for constructing efficient and stable all-inorganic CsPbI_2.2_Br_0.8_ PSCs under ambient conditions. It was found that the FABr additive plays a vital role in retarding crystal growth during the crystallization process, resulting in well-ordered crystal growth, enhanced crystallinity, highly dense and smooth morphology, prolonged carrier lifetime, decreased bulk and surface defects, suppressed trap-assisted recombination, and mitigated energy losses. As a result of these beneficial properties, the FABr-0.8%-based PSC showed a decent *PCE* of 17.95%, which was higher than that of the control device (15.94%). Moreover, the FABr-0.8%-assisted PSC exhibited higher long-term thermal stability by retaining~72.30% of the original *PCE*, compared to the control device that maintained 35.11% of the initial value after aging at 85 °C for 600 h under air conditions (RH ~10–20%, temperature ~20–28 °C). Furthermore, the FABr-0.8%-based PSC demonstrated an impressive indoor *PCE* of 31.22%, which was higher than control device (23.15%) under LED lighting conditions (3200 K, 1000 lux). Thus, the present work provides a feasible approach to develop a high-quality CsPbI_2.2_Br_0.8_ perovskite film under ambient conditions that will promote further development of efficient all-inorganic PSCs.

## Figures and Tables

**Table 1:** Photovoltaic parameters (reverse-bias condition) of control and FABr-based PSCs at different concentrations (0.4, 0.8, and 1.2M%).

Devices		*J_SC_* (mA/cm^2^)	*V_OC_* (mV)	*FF* (%)	*PCE* (%)
Control	Champion	16.10	1230	80.51	15.94
	Average	15.81 ± 0.29	1212 ± 18	79.50 ±1.01	15.23 ± 0.81
FABr-0.4M%	Champion	16.42	1265	82.44	17.12
	Average	16.21 ± 0.23	1250 ± 15	81.70 ± 0.74	16.55 ± 0.57
FABr-0.8M%	Champion	16.46	1293	84.36	17.95
	Average	16.31 ± 0.15	1283 ± 10	83.80 ± 0.56	17.54 ± 0.42
FABr-1.2M%	Champion	16.33	1224	81.22	16.23
	Average	16.08 ± 0.25	1205 ± 19	80.37 ± 0.85	15.57 ± 0.66

**Table 2:** Indoor *PV* parameters of PSCs (control and FABr-0.8M%).

Devices	*J_SC_* (μA/cm^2^)	*V_OC_* (mV)	*FF* (%)	*P_indoor_* (μW/cm^2^)	*PCE* (%)
Control	169	980	53.12	88	23.15
FABr-0.8M%	201	1031	57.27	118.7	31.22

## Data Availability

Data will be made available on request to the corresponding author.

## Supplementary Materials

The following supporting information can be downloaded at https://www.mdpi.com/article/10.3390/nano13192716/s1: Figure S1: Schematic demonstration of fabrication procedure of perovskite solar cells; Figure S2: XPS patterns of (a) Cs 3d, (b) Pb 4f, (c) I 3d, (d) Br 3d, and (e) N 1s of 100 °C and 240 °C FABr-0.8M% annealed perovskite films. Figure S3: XPS patterns of (a) Cs 3d, (b) Pb 4f, (c) I 3d, (d) Br 3d, and (e) N 1s of 240 °C FABr-0.8M% annealed perovskite film and after etching the surface through Ar ion etching gun for 20 sec. Figure S4: JV curves of control, FABr-0.4M%-, FABr-0.8M%-, and FABr-1.2M%-based PSCs; Figure S5: PCE distribution histograms for control and FABr-0.8M%-based PSCs; Figure S6: Energy loss corresponding to control and FABr-0.8M%-based PSCs. Figure S7: (a) Power consumption of various electronic devices and (b) electronic applications for indoor perovskite solar cells; Figure S8: JV curves of optimized PSC at (a) 1500 lux and (b) 2000 lux. The inset table shows the corresponding indoor photovoltaic parameters; Table S1: Reported research studies on CsPb_2.25_Br_0.75_ perovskite composition; Table S2: Crystal lattice parameters corresponding to (100) and (200) planes for control and FABr-0.8M% perovskite thin films; Table S3: TRPL fitted parameters for control and FABr-0.8M% perovskite thin films. The TRPL parameters were fitted using the biexponential decay model; Table S4: Photovoltaic parameters of control and FABr-based PSCs using different concentrations (0.4, 0.8, and 1.2M%); Table S5: Nyquist parameters of the control and FABr-0.8M%-based PSCs. References [19,21,65,66,67] are cited in the Supplementary Materials.

## References

[1] Lee D.S., Yun J.S., Kim J., Soufiani A.M., Chen S., Cho Y., Deng X., Seidel J., Lim S., Huang S. (2018). Passivation of grain boundaries by phenethylammonium in formamidinium-methylammonium lead halide perovskite solar cells. ACS Energy Lett..

[2] Thakur U.K., Kumar P., Gusarov S., Kobryn A.E., Riddell S., Goswami A., Alam K.M., Savela S., Kar P., Thundat T. (2020). Consistently High V oc Values in pin Type Perovskite Solar Cells Using Ni^3+^-Doped NiO Nanomesh as the Hole Transporting Layer. ACS Appl. Mater. Interfaces.

[3] Jiang J., Li R., Liu D., Xie H., Zeng Q., Li Y. (2023). Efficient and Stable CsPbI2Br Inorganic Perovskite Solar Cell Co-Modified with Ionic Liquids and Quantum Dots. ACS Appl. Energy Mater..

[4] Wang H., Bian H., Jin Z., Zhang H., Liang L., Wen J., Wang Q., Ding L., Liu S.F. (2019). Cesium lead mixed-halide perovskites for low-energy loss solar cells with efficiency beyond 17%. Chem. Mater..

[5] Kojima A., Teshima K., Shirai Y., Miyasaka T. (2009). Organometal halide perovskites as visible-light sensitizers for photovoltaic cells. J. Am. Chem. Soc..

[6] Park J., Kim J., Yun H.-S., Paik M.J., Noh E., Mun H.J., Kim M.G., Shin T.J., Seok S.I. (2023). Controlled growth of perovskite layers with volatile alkylammonium chlorides. Nature.

[7] Arora N., Dar M.I., Hinderhofer A., Pellet N., Schreiber F., Zakeeruddin S.M., Grätzel M. (2017). Perovskite solar cells with CuSCN hole extraction layers yield stabilized efficiencies greater than 20%. Science.

[8] Zhao X., Liu T., Burlingame Q.C., Liu T., Holley R., Cheng G., Yao N., Gao F., Loo Y.-L. (2022). Accelerated aging of all-inorganic, interface-stabilized perovskite solar cells. Science.

[9] Duan C., Wen Q., Fan Y., Li J., Liu Z., Yan K. (2022). Improving the stability and scalability of all-inorganic inverted CsPbI_2_Br perovskite solar cell. J. Energy Chem..

[10] Zheng S., Wang H., Wei P., Chen H., Xie Y. (2021). Enhancing the performance and stability of carbon-based CsPbI_2_Br perovskite solar cells via tetrabutylammonium iodide surface passivation. Sol. Energy.

[11] Zheng Y., Yang X., Su R., Wu P., Gong Q., Zhu R. (2020). High-performance CsPbI_x_Br_3_-x all-inorganic perovskite solar cells with efficiency over 18% via spontaneous interfacial manipulation. Adv. Funct. Mater..

[12] Zeng Q., Zhang X., Liu C., Feng T., Chen Z., Zhang W., Zheng W., Zhang H., Yang B. (2019). Inorganic CsPbI_2_Br perovskite solar cells: The progress and perspective. Sol. Rrl.

[13] Liu X., Jiang J., Wang F., Xiao Y., Sharp I.D., Li Y. (2019). High photovoltage inverted planar heterojunction perovskite solar cells with all-inorganic selective contact layers. ACS Appl. Mater. Interfaces.

[14] Lau C.F.J., Wang Z., Sakai N., Zheng J., Liao C.H., Green M., Huang S., Snaith H.J., Ho-Baillie A. (2019). Fabrication of efficient and stable CsPbI_3_ perovskite solar cells through cation exchange process. Adv. Energy Mater..

[15] Liu C., Yang Y., Xia X., Ding Y., Arain Z., An S., Liu X., Cristina R.C., Dai S., Nazeeruddin M.K. (2020). Soft template-controlled growth of high-quality CsPbI3 films for efficient and stable solar cells. Adv. Energy Mater..

[16] Chen W., Zhang J., Xu G., Xue R., Li Y., Zhou Y., Hou J., Li Y. (2018). A semitransparent inorganic perovskite film for overcoming ultraviolet light instability of organic solar cells and achieving 14.03% efficiency. Adv. Mater..

[17] Han Q., Yang S., Wang L., Yu F., Zhang C., Wu M., Ma T. (2021). The sulfur-rich small molecule boosts the efficiency of carbon-based CsPbI_2_Br perovskite solar cells to approaching 14%. Sol. Energy.

[18] Yu L., Guo T., Yuan H., Zhang Z., Deng Z., Zhao R., Zheng M., Zhang J., Xu W., Liu X. (2021). Effective lewis base additive with S-donor for efficient and stable CsPbI_2_Br based perovskite solar cells. Chem. Eng. J..

[19] Fang Z., Liu L., Zhang Z., Yang S., Liu F., Liu M., Ding L. (2019). CsPbI_2.25_Br_0.75_ solar cells with 15.9% efficiency. Sci. Bull..

[20] Zhang J., Wang Z., Mishra A., Yu M., Shasti M., Tress W., Kubicki D.J., Avalos C.E., Lu H., Liu Y. (2020). Intermediate phase enhances inorganic perovskite and metal oxide interface for efficient photovoltaics. Joule.

[21] Duan L., Wang Z., Li Y., Tan L., Zhang Z., Wang H., Yi C., Hagfeldt A., Luo J. (2021). Hydrophobic organic ammonium halide modification toward highly efficient and stable CsPbI_2.25_Br_0.75_ solar cell. Sol. Rrl.

[22] Bahadur J., Ryu J., Pandey P., Cho S., Cho J.S., Kang D.-W. (2023). In situ crystal reconstruction strategy-based highly efficient air-processed inorganic CsPbI_2_Br perovskite photovoltaics for indoor, outdoor, and switching applications. Nanoscale.

[23] Chen S.-C., Wang D., Zheng Q. (2020). Surface Passivation of All-Inorganic CsPbI_2_Br with a Fluorinated Organic Ammonium Salt for Perovskite Solar Cells with Efficiencies over 16%. Sol. RRL.

[24] Mali S.S., Patil J.V., Shinde P.S., de Miguel G., Hong C.K. (2021). Fully air-processed dynamic hot-air-assisted M: CsPbI_2_Br (M: Eu^2+^, In^3+^) for stable inorganic perovskite solar cells. Matter.

[25] Dong C., Han X., Zhao Y., Li J., Chang L., Zhao W. (2018). A green anti-solvent process for high performance carbon-based CsPbI_2_Br all-inorganic perovskite solar cell. Solar RRL.

[26] Liu C., Li W., Li H., Wang H., Zhang C., Yang Y., Gao X., Xue Q., Yip H.L., Fan J. (2019). Structurally reconstructed CsPbI_2_Br perovskite for highly stable and square-centimeter all-inorganic perovskite solar cells. Adv. Energy Mater..

[27] Wang K., Zhou J., Li X., Ahmad N., Xia H., Wu G., Zhang X., Wang B., Zhang D., Zou Y. (2020). A surface modifier enhances the performance of the all-inorganic CsPbI_2_Br perovskite solar cells with efficiencies approaching 15%. Phys. Chem. Chem. Phys..

[28] Tian J., Zhang K., Xie Z., Peng Z., Zhang J., Osvet A., Lüer L., Kirchartz T., Rau U., Li N. (2022). Quantifying the Energy Losses in CsPbI_2_Br Perovskite Solar Cells with an Open-Circuit Voltage of up to 1.45 V. ACS Energy Lett..

[29] Zhou Q., Cai C., Xiong Q., Zhang Z., Xu J., Liang L., Wang S., Sun W., Yuan Z., Gao P. (2022). Surface Polarity Regulation by Relieving Fermi-Level Pinning with Naphthalocyanine Tetraimides toward Efficient Perovskite Solar Cells with Improved Photostability. Adv. Energy Mater..

[30] Li D., Huang Y., Ma R., Liu H., Liang Q., Han Y., Ren Z., Liu K., Fong P.W.K., Zhang Z. (2023). Surface Regulation with Polymerized Small Molecular Acceptor towards Efficient Inverted Perovskite Solar Cells. Adv. Energy Mater..

[31] Yue X., Yang Y., Zhao X., Fan B., Yan H., Qu S., Zhang Q., Lan Z., Du S., Huang H. (2023). In situ surface regulation of 3D perovskite using diethylammonium iodide for highly efficient perovskite solar cells. Phys. Chem. Chem. Phys..

[32] Li X., Ying Z., Zheng J., Wang X., Chen Y., Wu M., Xiao C., Sun J., Shou C., Yang Z. (2023). Surface Reconstruction for Efficient and Stable Monolithic Perovskite/Silicon Tandem Solar Cells with Greatly Suppressed Residual Strain. Adv. Mater..

[33] Xu Y., Ren Y., Cheng S., Zhang L., Niu P., Lyu M., Lu H., Wang M., Zhu J. (2023). A residual strain regulation strategy based on quantum dots for efficient perovskite solar cells. J. Mater. Chem. A.

[34] Mohanta M.K., Is F., Kishore A., De Sarkar A. (2021). Spin-current modulation in hexagonal buckled ZnTe and CdTe monolayers for self-powered flexible-piezo-spintronic devices. ACS Appl. Mater. Interfaces.

[35] Patil J.V., Mali S.S., Hong C.K. (2022). Reducing defects of all-inorganic γ-CsPbI_2_Br thin films by ethylammonium bromide additives for efficient perovskite solar cells. ACS Appl. Mater. Interfaces.

[36] Bahadur J., Ryu J., Lee D.-G., Hong J., Hayase S., Cho J.S., Jeong S.M., Kang D.-W. (2023). In-situ surface defects passivation with small carbon chain molecules for highly efficient, air-processed inorganic CsPbI_2_Br perovskite photovoltaics. Appl. Surf. Sci..

[37] Zhang C., Wan X., Zang J., Liu Q., Fei Y., Yu Z. (2021). Polymer-modified CsPbI_2_Br films for all-inorganic planar perovskite solar cells with improved performance. Surf. Interfaces.

[38] Jin I.S., Parida B., Jung J.W. (2022). Simultaneously enhanced efficiency and ambient stability of inorganic perovskite solar cells by employing tetramethylammonium chloride additive in CsPbI_2_Br. J. Mater. Sci. Technol..

[39] Li H., Hao X., Chang B., Li Z., Wang L., Pan L., Chen X., Yin L. (2021). Stiffening the Pb-X framework through a π-conjugated small-molecule cross-linker for high-performance inorganic CsPbI_2_Br perovskite solar cells. ACS Appl. Mater. Interfaces.

[40] Fu S., Wang J., Liu X., Yuan H., Xu Z., Long Y., Zhang J., Huang L., Hu Z., Zhu Y. (2021). Multifunctional liquid additive strategy for highly efficient and stable CsPbI_2_Br all-inorganic perovskite solar cells. Chem. Eng. J..

[41] Bahadur J., Cho S., Pandey P., Ryu J., Yoon S., Lee D.-G., Song J.T., Cho J.S., Kang D.-W. (2023). Surface defect passivation of All-Inorganic CsPbI_2_Br perovskites via fluorinated ionic liquid for efficient Outdoor/Indoor photovoltaics processed in ambient air. Appl. Surf. Sci..

[42] Mali S.S., Patil J.V., Hong C.K. (2019). Hot-air-assisted fully air-processed barium incorporated CsPbI_2_Br perovskite thin films for highly efficient and stable all-inorganic perovskite solar cells. Nano Lett..

[43] Wu N., Yang T., Wang Z., Wu Y., Wang Y., Ma C., Li H., Du Y., Zhao D., Wang S. (2023). Stabilizing precursor solution and controlling crystallization kinetics simultaneously for high-performance perovskite solar cells. Adv. Mater..

[44] Ma R., Li H., Peña T.A.D., Xie X., Fong P.W.K., Wei Q., Yan C., Wu J., Cheng P., Li M. (2023). Tunable Donor Aggregation Dominance in Ternary Matrix of All-polymer Blends with Improved Efficiency and Stability. Adv. Mater..

[45] Lee J.W., Lim C., Lee S.W., Jeon Y., Lee S., Kim T.S., Lee J.Y., Kim B.J. (2022). Intrinsically Stretchable and Non-Halogenated Solvent Processed Polymer Solar Cells Enabled by Hydrophilic Spacer-Incorporated Polymers. Adv. Energy Mater..

[46] Li Y., Duan J., Yuan H., Zhao Y., He B., Tang Q. (2018). Lattice modulation of alkali metal cations doped Cs_1−x_R_x_PbBr_3_ halides for inorganic perovskite solar cells. Sol. RRL.

[47] Zhang W., Xiong J., Li J., Daoud W.A. (2020). Guanidinium Passivation for Air-Stable Rubidium-Incorporated Cs_(1−x)_Rb_x_PbI_2_Br Inorganic Perovskite Solar Cells. Solar RRL.

[48] Patil J.V., Mali S.S., Hong C.K. (2022). Grain size enlargement and controlled crystal growth by formamidinium chloride additive-added γ-CsPbI_2_Br thin films for stable inorganic perovskite solar cells. Mater. Today Chem..

[49] Khalid S., Malik M.A., Lewis D.J., Kevin P., Ahmed E., Khan Y., O’Brien P. (2015). Transition metal doped pyrite (FeS_2_) thin films: Structural properties and evaluation of optical band gap energies. J. Mater. Chem. C.

[50] Mote V., Purushotham Y., Dole B. (2012). Williamson-Hall analysis in estimation of lattice strain in nanometer-sized ZnO particles. J. Theor. Appl. Phys..

[51] Fu S., Zhang W., Li X., Wan L., Wu Y., Chen L., Liu X., Fang J. (2020). Dual-protection strategy for high-efficiency and stable CsPbI_2_Br inorganic perovskite solar cells. ACS Energy Lett..

[52] Li H., Yin L. (2020). Efficient Bidentate Molecules Passivation Strategy for High-Performance and Stable Inorganic CsPbI_2_Br Perovskite Solar Cells. Sol. RRL.

[53] Grischek M., Caprioglio P., Zhang J., Pena-Camargo F., Sveinbjörnsson K., Zu F., Menzel D., Warby J.H., Li J., Koch N. (2022). Efficiency Potential and Voltage Loss of Inorganic CsPbI_2_Br Perovskite Solar Cells. Sol. RRL.

[54] Jin Z., Yuan M., Li H., Yang H., Zhou Q., Liu H., Lan X., Liu M., Wang J., Sargent E.H. (2016). Graphdiyne: An efficient hole transporter for stable high-performance colloidal quantum dot solar cells. Adv. Funct. Mater..

[55] Niu G., Li W., Meng F., Wang L., Dong H., Qiu Y. (2014). Study on the stability of CH_3_NH_3_PbI_3_ films and the effect of post-modification by aluminum oxide in all-solid-state hybrid solar cells. J. Mater. Chem. A.

[56] Guerrero A., Bisquert J., Garcia-Belmonte G. (2021). Impedance spectroscopy of metal halide perovskite solar cells from the perspective of equivalent circuits. Chem. Rev..

[57] Yang Y., Song J., Zhao Y., Zhu L., Gu X., Gu Y., Che M., Qiang Y. (2016). Ammonium-iodide-salt additives induced photovoltaic performance enhancement in one-step solution process for perovskite solar cells. J. Alloys Compd..

[58] Song J., Li S., Zhao Y., Yuan J., Zhu Y., Fang Y., Zhu L., Gu X., Qiang Y. (2017). Performance enhancement of perovskite solar cells by doping TiO_2_ blocking layer with group VB elements. J. Alloys Compd..

[59] Bi Z., Xu X., Chen X., Zhu Y., Liu C., Yu H., Zheng Y., Troshin P.A., Guerrero A., Xu G. (2022). High-performance large-area blade-coated perovskite solar cells with low ohmic loss for low lighting indoor applications. Chem. Eng. J..

[60] Sha W.E., Zhang H., Wang Z.S., Zhu H.L., Ren X., Lin F., Jen A.K.Y., Choy W.C. (2018). Quantifying efficiency loss of perovskite solar cells by a modified detailed balance model. Adv. Energy Mater..

[61] Chen F.C. (2019). Emerging organic and organic/inorganic hybrid photovoltaic devices for specialty applications: Low-level-lighting energy conversion and biomedical treatment. Adv. Opt. Mater..

[62] Virtuani A., Lotter E., Powalla M. (2003). Performance of Cu (In, Ga) Se_2_ solar cells under low irradiance. Thin Solid Film..

[63] Vincent P., Shin S.-C., Goo J.S., You Y.-J., Cho B., Lee S., Lee D.-W., Kwon S.R., Chung K.-B., Lee J.-J. (2018). Indoor-type photovoltaics with organic solar cells through optimal design. Dye. Pigment..

[64] Lee J.W., Park N.G. (2020). Chemical approaches for stabilizing perovskite solar cells. Adv. Energy Mater..

[65] Habisreutinger S.N., Noel N.K., Snaith H.J. (2018). Hysteresis index: A figure without merit for quantifying hysteresis in perovskite solar cells. ACS Energy Lett..

[66] Fang Z., Meng X., Zuo C., Li D., Xiao Z., Yi C., Wang M., Jin Z., Yang S., Ding L. (2019). Interface engineering gifts CsPbI2. 25Br0. 75 solar cells high performance. Sci. Bull..

[67] Xiao H., Zuo C., Zhang L., Zhang W., Hao F., Yi C., Liu F., Jin H., Ding L. (2023). Efficient inorganic perovskite solar cells made by drop-coating in ambient air. Nano Energy.

