# Supplementary material for: Controlled Crystal Growth of All-Inorganic CsPbI2.2Br0.8 Thin Film via Additive Strategy for Air-Processed Efficient Outdoor/Indoor Perovskite Solar Cells
Source: Nanomaterials (Basel). 2023 Oct 6;13(19):2716. doi: 10.3390/nano13192716 (PMC10574290; doi:10.3390/nano13192716)
Supplement: Supplementary file 1 [file nanomaterials-13-02716-s001.zip › nanomaterials-2620620-supplementary.pdf]

*Supplementary Materials*

# Controlled Crystal Growth of All-Inorganic $CsPbI_{2.2}Br_{0.8}$ Thin Film via Additive Strategy for Air-Processed Efficient Outdoor/Indoor Perovskite Solar Cells

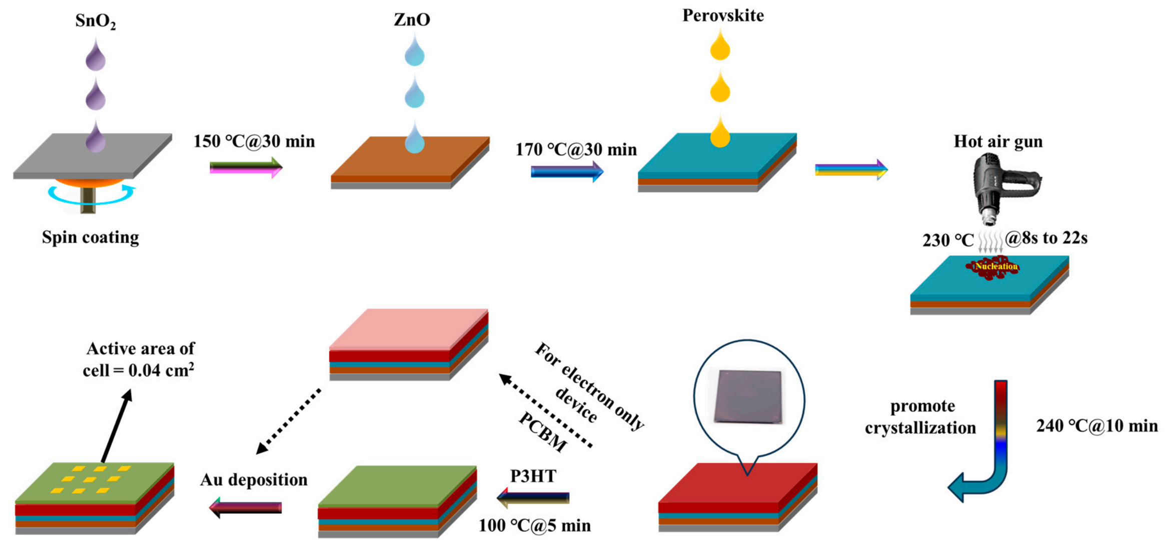


**Fig. S1** Schematic demonstration of fabrication procedure of perovskite solar cells.

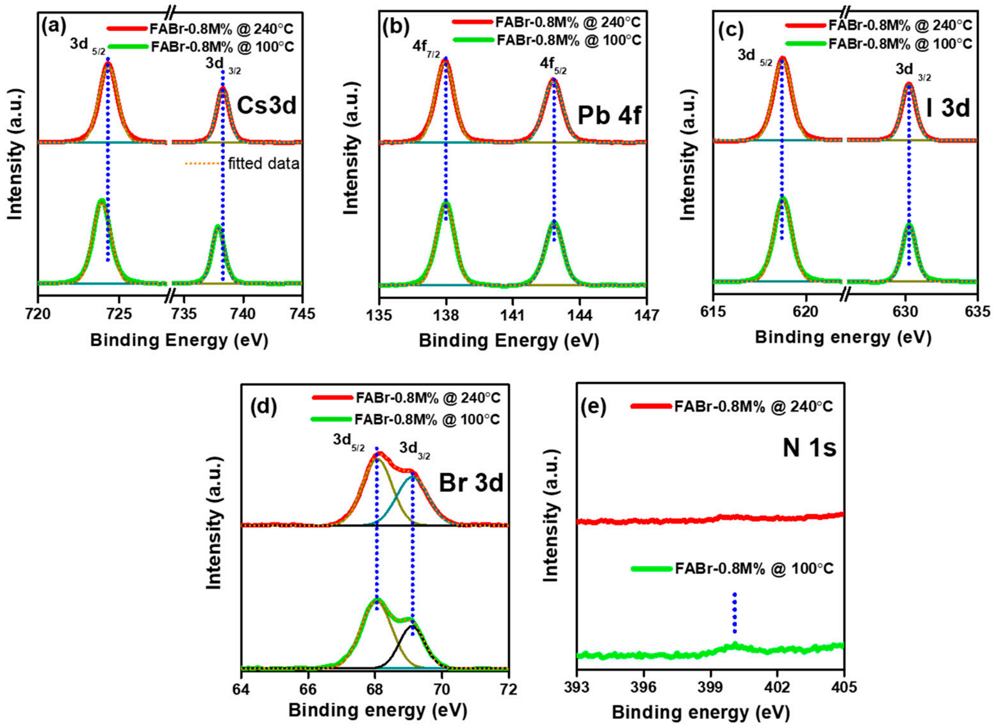


**Fig. S2** XPS patterns of (a) Cs 3d, (b) Pb 4f, (c) I 3d, (d) Br 3d, and (e) N 1s of 100 °C and 240 °C FABr-0.8M% annealed perovskite films.

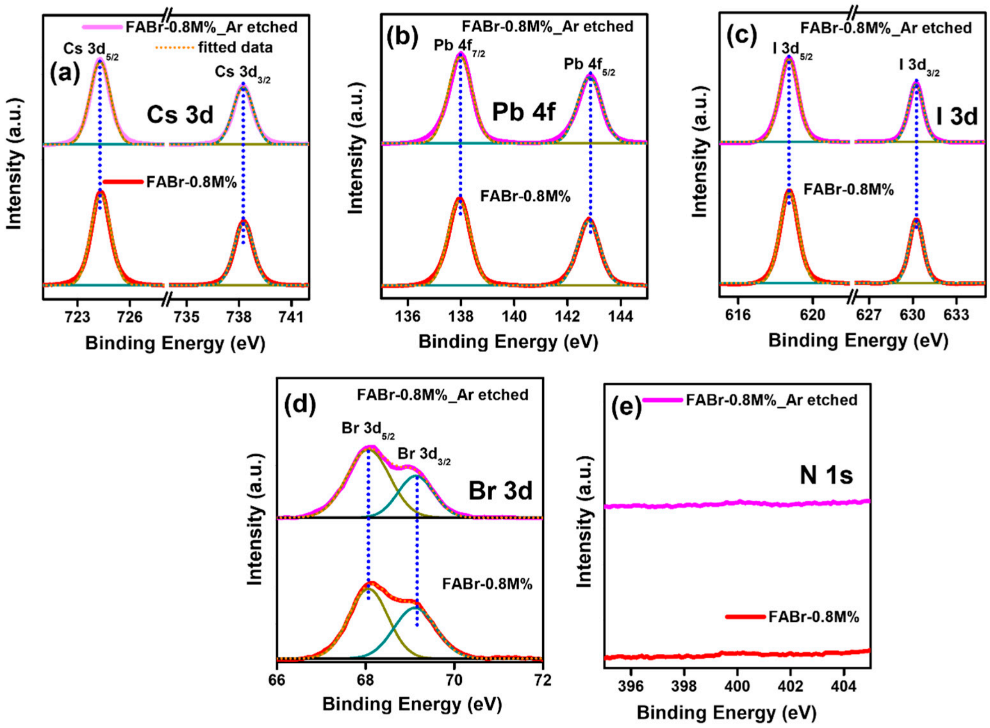


**Fig. S3** XPS patterns of (a) Cs 3d, (b) Pb 4f, (c) I 3d, (d) Br 3d, and (e) N 1s of 240 °C FABr-0.8M% annealed perovskite film and after etching the surface through Ar ion etching gun.

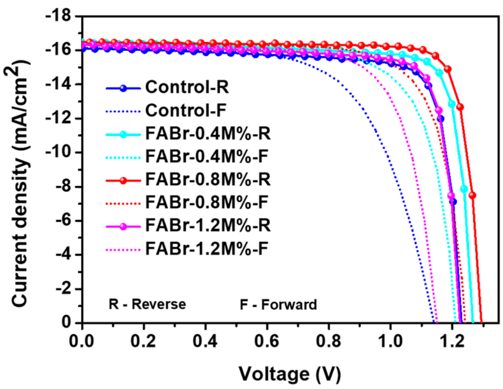


**Fig. S4** JV curves of control, FABr-0.4M%, FABr-0.8M% and FABr-1.2M% based PSCs.

The hysteresis index (*HI*) was calculated using the following formula [S1] and obtained values were tabulated in Table S4.

$$HI = \frac{PCE_{(reverse)} - PCE_{(forward)}}{\mathrm{PCE}_{(reverse)}}$$

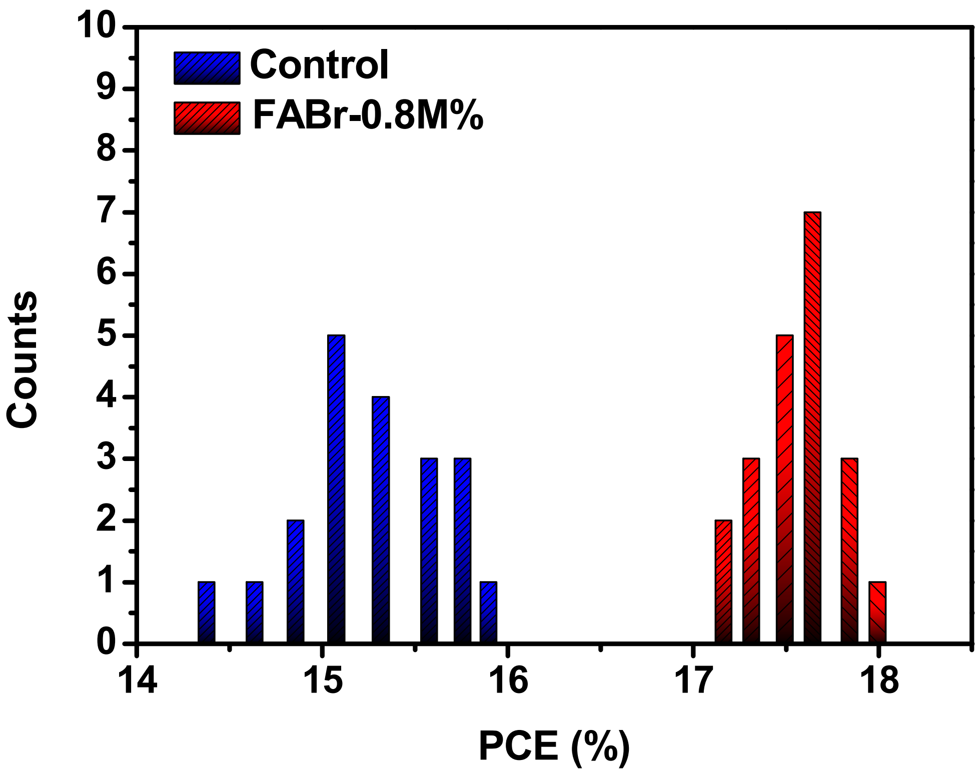


**Fig. S5** PCEs distribution histograms for control and FABr-0.8M% based PSCs.

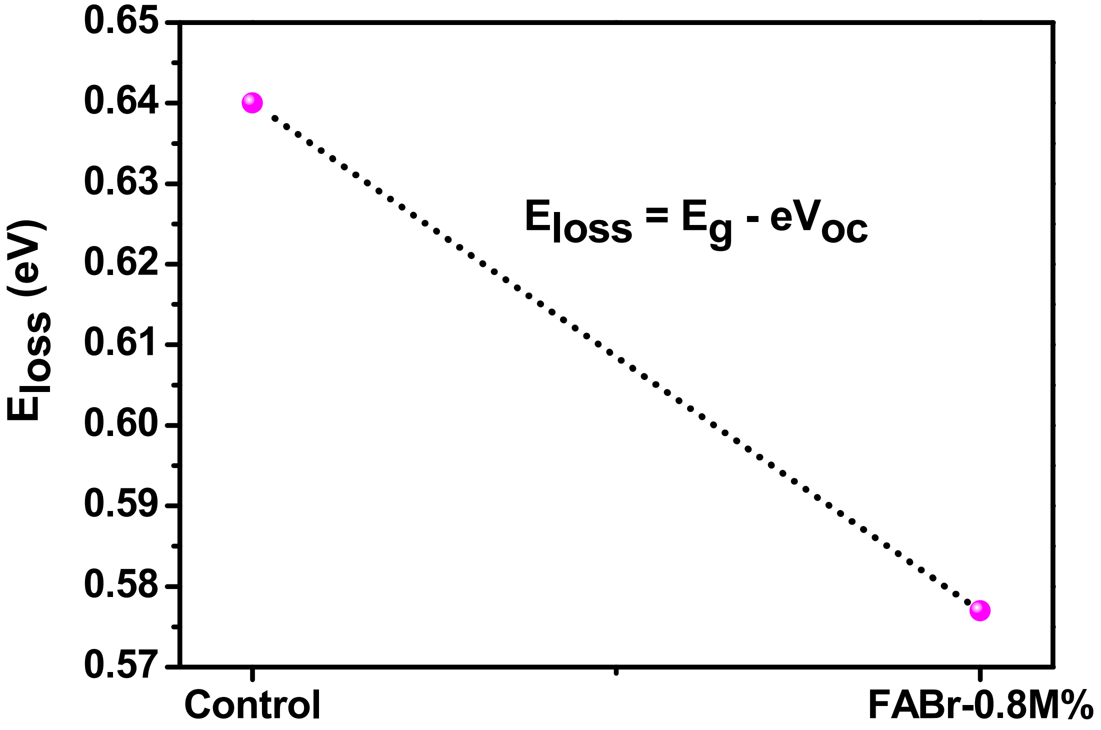


**Fig. S6** Energy loss corresponding to control and FABr-0.8M% based PSCs.

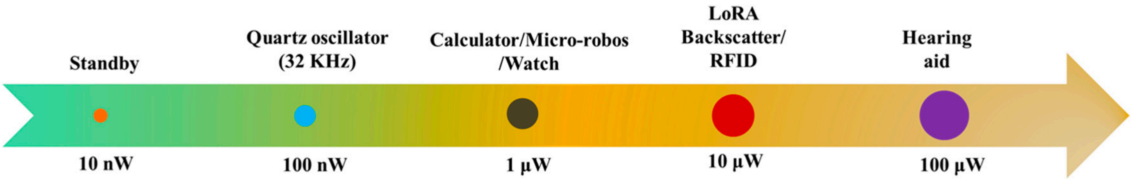


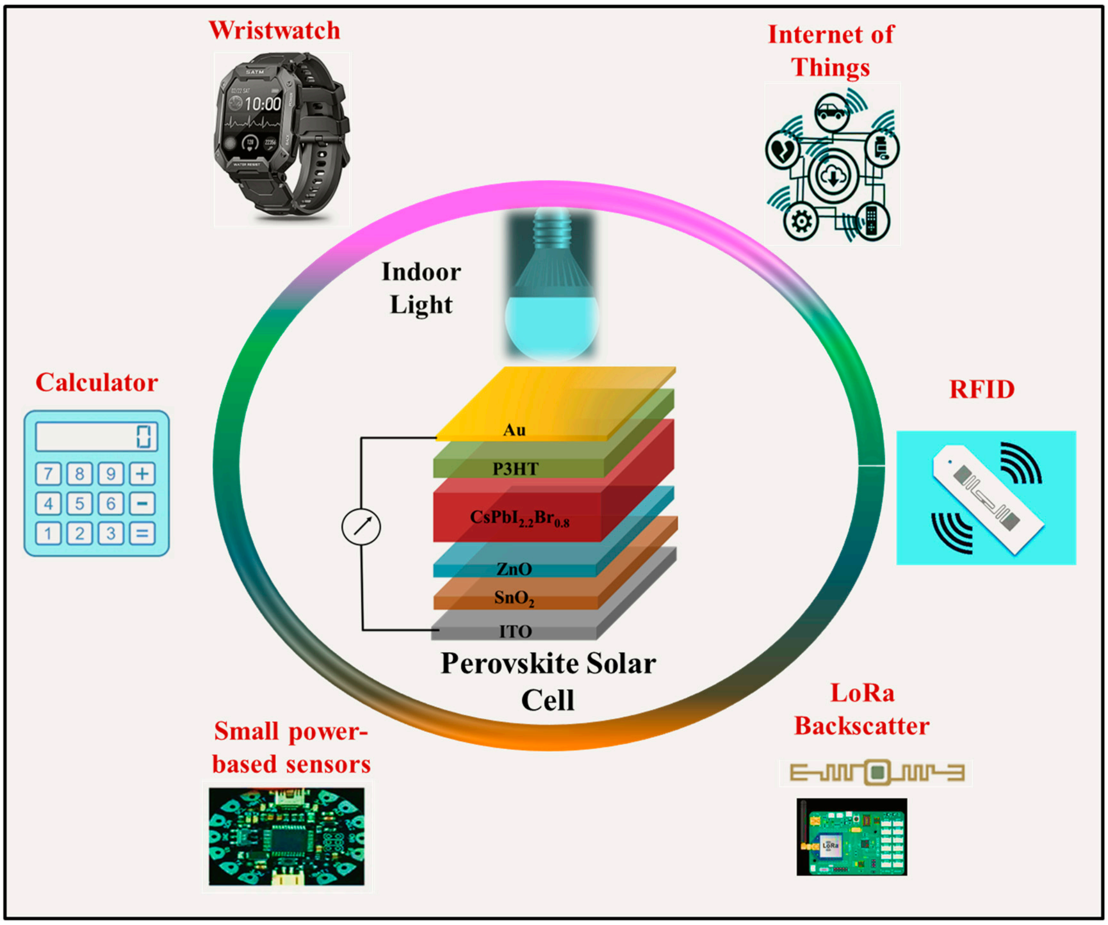


**Fig. S7 (a)** Power consumption of various electronic devices, and **(b)** Electronic applications for indoor perovskite solar cells.

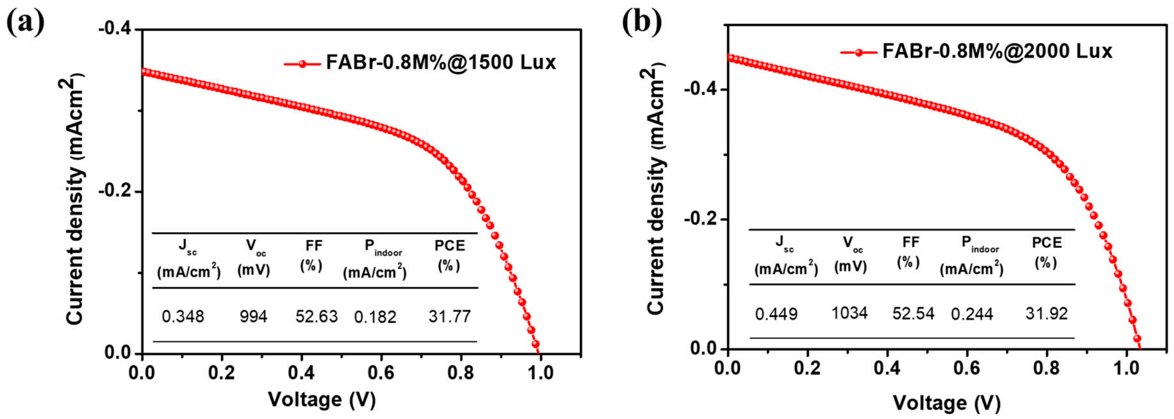


Fig. S8: JV curves of optimized PSC at (a) 1500 lux and (b) 2000 lux. The inset table shows the corresponding indoor photovoltaic parameters.

**Table S1** Reported research studies on $CsPb_{2.25}Br_{0.75}$ perovskite composition.

| **Reference with device structure** | **Methodology** | **Novelty Statement** | **Processing conditions** | **PCE (%)** | |
|---|---|---|---|---|---|
| | | | | **Out-door** | **In-door** |
| $ITO/ZnO{:}DPPA/CsPbI_{2.25}Br_{0.75}/PTAA/MoO_3/Au$ [S2] | Surface modification of ETL layer | Authors suggested that ZnO surface defects significantly reduced with DPAA treatment, which is beneficial to formation of high-quality perovskite film. | $N_2$-filled glovebox | 15.90 | - |
| $ITO/SnO_2/$ $CsPb(I_{0.75}Br_{0.25})_3$-0.5FAAc:CTAC/ Spiro-OMeTAD/Au [S3] | Surface passivation strategy | Authors demonstrated that hexadecyltrimethylammonium chloride (CTAC) treatment efficiently passivated the perovskite surface, resulting in improved the device PCE as well as stability. | RH-10% | 18.05 | - |

| | | | | | |
|---|---|---|---|---|---|
| $ITO/SnO_2/ZnO/CsPbI_{2.25}Br_{0.75}/$ $PBD2T:PTAA/MoO_3/Ag$ [S4] | Additive strategy with HTL layer | Authors suggested that addition of donor polymer PBD2T into PTAA facilitate suitable band energy alignment corresponding to perovskite, resulting in reduced energy losses. | $N_2$ filled glovebox | 17.37 | - |
| $ITO/SnO_2/ZnO/CsPbI_{2.25}Br_{0.75}$: $Pb(Ac)_2.3H_2O/PTAA/MoO_3/Ag$ [S5] | Drop-coating strategy | Authors suggested that lead acetate trihydrate as an additive into perovskite precursor and airflow drying controlled the nucleation and crystal growth, leading to high quality of perovskite film. | Ambient conditions | 18.49 | - |
| **<This work>** | | | | | |
| $ITO/SnO_2/ZnO/CsPbI_{2.2}Br_{0.8}$:FA Br /P3HT/Au | Additive strategy with perovskite composition | Authors demonstrated that FABr as an additive plays key role to retard the crystallization kinetics and control the crystal growth by limiting the formation of nuclei centers, resulting in improved film quality. The FABr assisted device showed high PCE as well as good long-term stability as compared to control PSC. | Ambient air (RH ~ 30-40%) | 17.95 | 31.22 |

**Table S2** Crystal lattice parameters of corresponding to (100) and (200) planes for control and FABr-0.8M% perovskite thin films.

| **Parameters** | **Control (100)** | **FABr-0.8M% (100)** | **Control (200)** | **FABr-0.8M% (200)** | **Average** | |
|---|---|---|---|---|---|---|
| | | | | | **Control** | **FABr-0.8M%** |
| Full width at half maximum (*β*, ˚) | 0.1775 | 0.1126 | 0.2085 | 0.1860 | 0.1930 | 0.1493 |
| Peak angle (2*θ*, ˚) | 14.72 | 14.70 | 29.58 | 29.57 | - | |
| Average crystallite size (*D*, nm) | 45.12 | 71.13 | 39.40 | 44.10 | 42.26 | 57.61 |
| Strain (*ε*) | 0.2834 | 0.1802 | 0.1629 | 0.1450 | 0.2231 | 0.1626 |
| Dislocation density (*δ*, nm$^{-2}$) | - | - | - | - | $5.56\times10^{-4}$ | $3.01\times10^{-4}$ |

**Table S3** TRPL fitted parameters for control and FABr-0.8M% perovskite thin films. The TRPL parameters was fitted using the biexponential decay model.

| **Perovskites** | **$\tau_1$(ns)** | **$A_1$ (%)** | **$\tau_2$(ns)** | **$A_2$ (%)** | **$\tau_{avg}$ (ns)** |
|---|---|---|---|---|---|
| Control | 29.90 | 18.73 | 87.49 | 81.27 | 83 |
| FABr-0.8M% | 49.65 | 19.34 | 132.71 | 80.66 | 125 |

**Table S4** Photovoltaic parameters of control and FABr different concentrations (0.4, 0.8, and 1.2M%) based PSCs.

<table>
<tr><th>Devices</th><th>$J_{SC}$ (mA/cm$^2$)</th><th>$V_{OC}$ (mV)</th><th>FF (%)</th><th>FF (%)</th><th>PCE (%)</th><th>HI (%)</th></tr>
<tr><td>Control-R</td><td>16.10</td><td>1230</td><td>80.51</td><td>80.51</td><td>15.94</td><td rowspan="2">26.16</td></tr>
<tr><td>Control-F</td><td>16.15</td><td>1140</td><td>63.94</td><td>63.94</td><td>11.77</td></tr>
<tr><td>FABr-0.4M%-R</td><td>16.42</td><td>1265</td><td>82.44</td><td>82.44</td><td>17.12</td><td rowspan="2">15.47</td></tr>
<tr><td>FABr-0.4M%-F</td><td>16.43</td><td>1209</td><td>72.85</td><td>72.85</td><td>14.47</td></tr>
<tr><td>FABr-0.8M%-R</td><td>16.46</td><td>1293</td><td>84.36</td><td>84.36</td><td>17.95</td><td rowspan="2">14.59</td></tr>
<tr><td>FABr-0.8M%-F</td><td>16.46</td><td>1241</td><td>75.05</td><td>75.05</td><td>15.33</td></tr>
<tr><td>FABr-1.2M%-R</td><td>16.33</td><td>1224</td><td>81.22</td><td>81.22</td><td>16.23</td><td rowspan="2">15.71</td></tr>
<tr><td>FABr-1.2M%-F</td><td>16.38</td><td>1150</td><td>72.64</td><td>72.64</td><td>13.68</td></tr>
</table>

**Table S5** Nyquist parameters of the control and FABr-0.8M% based PSCs.

| Parameters | Control | FABr-0.8M% |
| --- | --- | --- |
| $R_s$ (Ω) | 55 | 43 |
| $R_{rec}$ (Ω) | 6478 | 9706 |

**References:**


S1. S. N. Habisreutinger, N. K. Noel and H. J. Snaith, Hysteresis index: A figure without merit for quantifying hysteresis in perovskite solar cells, *ACS Energy Letters* **2018**, *10*, 2472-2476.

S2. Z. Fang, L. Liu, Z. Zhang, S. Yang, F. Liu, M. Liu and L. Ding, CsPbI2. 25Br0. 75 solar cells with 15.9% efficiency, *Science bulletin* **2019**, *8*, 507-510.

S3. L. Duan, Z. Wang, Y. Li, L. Tan, Z. Zhang, H. Wang, C. Yi, A. Hagfeldt and J. Luo, Hydrophobic organic ammonium halide modification toward highly efficient and stable CsPbI2. 25Br0. 75 solar cell, *Solar Rrl* **2021**, *7*, 2100178.

S4. Z. Fang, X. Meng, C. Zuo, D. Li, Z. Xiao, C. Yi, M. Wang, Z. Jin, S. Yang and L. Ding, Interface engineering gifts CsPbI2. 25Br0. 75 solar cells high performance, *Science bulletin* **2019**, *23*, 1743-1746.

S5. H. Xiao, C. Zuo, L. Zhang, W. Zhang, F. Hao, C. Yi, F. Liu, H. Jin and L. Ding, Efficient inorganic perovskite solar cells made by drop-coating in ambient air, *Nano Energy* **2023**, 108061.
